# *Gli2*-Mediated *Shh* Signaling Is Required for Thalamocortical Projection Guidance

**DOI:** 10.3389/fnana.2022.830758

**Published:** 2022-02-10

**Authors:** Antuca Callejas-Marin, Juan Antonio Moreno-Bravo, Verónica Company, M. Pilar Madrigal, Francisca Almagro-García, Salvador Martínez, Eduardo Puelles

**Affiliations:** ^1^Instituto de Neurociencias, Consejo Superior de Investigaciones Científicas, Universidad Miguel Hernández de Elche, Elche, Spain; ^2^Departamento de Anatomía, Biología Celular y Zoología, Facultad de Ciencias, Universidad de Extremadura, Badajoz, Spain

**Keywords:** thalamocortical axons, guidance, *Gli2*, *Slit1*, *Slit2*

## Abstract

The thalamocortical projections are part of the most important higher level processing connections in the vertebrates and follow a highly ordered pathway from their origin in the thalamus to the cerebral cortex. Their functional complexities are not only due to an extremely elaborate axon guidance process but also due to activity-dependent mechanisms. *Gli2* is an intermediary transcription factor in the Sonic *hedgehog (Shh)* pathway. During neural early development, *Shh* has an important role in dorsoventral patterning, diencephalic anteroposterior patterning, and many later developmental processes, such as axon guidance and cell migration. Using a *Gli2* knockout mouse line, we have studied the role of *Shh* signaling mediated by *Gli2* in the development of the thalamocortical projections during embryonic development. In wild-type brains, we have described the normal trajectory of the thalamocortical axons into the context of the prosomeric model. Then, we have compared it with the altered thalamocortical axons course in *Gli2* homozygous embryos. The thalamocortical axons followed different trajectories and were misdirected to other territories probably due to alterations in the *Robo/Slit* signaling mechanism. In conclusion, the alteration of *Gli2-*mediated *Shh* signaling produces an erroneous specification of several territories related with the thalamocortical axons. This is translated into a huge modification in the pathfinding signaling mechanisms needed for the correct wiring of the thalamocortical axons.

## Introduction

The thalamus (Th) is a neuronal nuclei complex located in the diencephalon. Its functions involve sensory processing and regulation of motor functions as well as the control of sleep and awake states of consciousness (Jones, 2007). Also, as increasingly recognized, the Th acts as a link between distinct cortical areas (Theyel et al., 2010) and it is related to the cortical states control in a behaviorally relevant way (Cruikshank et al., 2010; Minlebaev et al., 2011; Poulet et al., 2012).

It develops from the alar portion neuroepithelium of prosomere 2 (Puelles et al., 1987; Puelles and Rubenstein, 1993, Puelles and Rubenstein, 2003) between embryonic days (E) E10.5 and E16.5 (Angevine, 1970; Puelles and Rubenstein, 1993, Puelles and Rubenstein, 2003). Primarily, it generates five pronuclei, which further differentiate into specific thalamic nuclei gradually building a complex nested structure (Jones, 2007). This structure is defined by the expression of the homeobox gene *Gbx2*, a transcription factor required for thalamic development (Miyashita-Lin et al., 1999). This region is a highly subdivided structure in terms of its nuclear organization. In mammals, this structure is constituted by dozens of morphologically and functionally distinct nuclei (Jones, 2007). In parallel with the process of progressive nuclear differentiation within the Th, there is a morphological and neurochemical differentiation of relay neurons, interneurons, and neuroglial cells, and finally, afferent and efferent connections are established (Jones, 2007). Its development is a model of the generation of an intricate, three-dimensional brain region from a two-dimensional neuroepithelium (Haddad-Tóvolli et al., 2012).

The thalamocortical projection constitutes one of the most prominent higher level processing connections in the mammalian brain. This projection conveys inputs to the sensory and motor cerebral cortex, where the processing of this information leads to perception and the organization of valid responses to internal and external stimuli. The innervation of the cerebral cortex by thalamic axons is a late event in brain development, but the thalamic axons growth toward their cortical targets begins quite early in embryogenesis even before the establishment of subcortical afferent connections to the Th (Molnár et al., 2012).

The thalamocortical projection functional complexity is due to a complex elaborate axon guidance process, linking the various thalamic nuclei with specific cortical regions (Castillo-Paterna et al., 2015; Mitsogiannis et al., 2017; López-Bendito, 2018; Nakagawa, 2019; Quintana-Urzainqui et al., 2020).

Around the second and third week of gestation (E12–E18 in mice), the cortex and Th start to link with each other through precise and reciprocal connections. Thalamocortical and corticothalamic projections have to cross diverse regions and morphogenetic boundary zones, including the diencephalic–prosencephalic, hypothalamic-telencephalic, and the pallial–subpallial boundaries, to reach their ultimate target cells. The thalamocortical axons (TCAs) follow a highly organized pathway from their Th origin to the cerebral cortex (López-Bendito and Molnár, 2003; Molnár et al., 2003, Molnár et al., 2012; Garel and Rubenstein, 2004). They move rostrally toward the alar peduncular hypothalamus (PHy), make a sharp turn dorsally at the alar PHy-telencephalic boundary to enter the mantle region of the medial ganglionic eminence (MGE), where they enter the internal capsule by E13 in mice, and then advance through the striatum, cross the pallial–subpallial boundaries, and finally reach the developing cortex.

It remains unclear how different thalamocortical projecting neural groups are topographically specified, and which transcription factors regulate the growth of their axons (López-Bendito and Molnár, 2003; Shimogori and Grove, 2005; Price et al., 2006; Quintana-Urzainqui et al., 2020). Numerous transcription factors are expressed in distinct but often overlapping patterns in the Th, suggesting that they must cooperate to control the specification and differentiation of thalamic nuclei and cell types. The zona limitans intrathalamica is the main organizer of the Th (Martinez-Ferre and Martinez, 2012). Its morphogenetic signal is *Sonic Hedgehog* (*Shh;* Scholpp and Lumsden, 2010). This morphogen induces the expression of transcription factors such as *Gbx2*, which is expressed broadly and early in the Th (Bulfone et al., 1993), and later it is required for the differentiation of a subset of nuclei and the development of TCA projections (Miyashita-Lin et al., 1999).

The Hedgehog family of secreted signaling molecules plays crucial roles in many aspects of embryonic development and adult tissue homeostasis [reviewed by Ingham and McMahon (2001) and Logan and Nusse (2004)]. Its role in the specification and differentiation of the diencephalic, an especially of the Th (Kiecker and Lumsden, 2004; Vieira et al., 2005; Vue et al., 2009; Jeong et al., 2011; Epstein, 2012; Ding et al., 2017).

Canonical *Shh* signaling occurs *via* a derepression mechanism involving the multiple-pass transmembrane receptor *Patched* (*Ptch*) and the G-protein-coupled receptor-like molecule *Smoothened* (*Smo*). *Shh* binding to *Ptch* relieves this repression, allowing *Smo* to translocate to the primary cilium where it activates intracellular signaling pathways. This activation of *Smo* culminates can activate or repress *Shh* target genes (Martí and Bovolenta, 2002).

In vertebrates, three *Gli* transcription factors (*Gli1*–*Gli3*) have been described, all of which are expressed in the developing neural tube (Hui et al., 1994; Lee et al., 1997; Ruiz i Altaba, 1998; Bai et al., 2002; Jacob and Briscoe, 2003). While *Gli2* and *Gli3* have transcriptional activator and repressor functions, *Gli1* is solely a transcriptional activator [reviewed by Ruiz i Altaba et al. (2007)]. Each *Gli* family member responds in a different way to *Shh*. In the absence of *Shh*, *Gli2* is fully degraded, while *Gli3* is converted (*via* proteolytic processing of its C-terminus) to a repressor of transcription (*Gli3R*). When *Shh* is present, *Gli1* expression is induced and proteolysis of *Gli2* and *Gli3* is inhibited, allowing full-length activator forms of the GLI proteins to accumulate. The *Gli* transcriptional activators then induce the expression of *Shh* pathway target genes (Dai et al., 1999; Ruiz I Altaba, 1999; Aza-Blanc et al., 2000; Pan et al., 2006). The *Gli2* loss of function analyzed in the spinal cord demonstrated a strong reduction in the generation the ventral fated neurons (Matise et al., 1998).

Our aim is to decipher the role of the *Shh-*induced mechanism mediated by *Gli2* in the development of the TCAs during embryonic development.

## Materials and Methods

### Animals

The transgenic mouse line *Gli2*^–/–^ was generated as previously described (Mo et al., 1997). The day when the vaginal plug was detected was considered as embryonic day 0.5 (E0.5). The embryos were fixed in phosphate buffered saline (PBS) 1x (NaCl 13 mM, Sigma S3014; KCl 0.3 mM, Sigma P9541; Na_2_HPO_4_ 1 mM, Sigma S3264 and KH_2_PO_4_ 0.2 mM, Sigma P9791) with 4% paraformaldehyde (PFA, Panreac 141451.1211) overnight at 4°C. The embryos were washed in PBS 1x, embedded in 4% agarose (Pronadisa 8008), and sectioned in 100 μm vibratome sections. For wax-embedded sections, the embryos were completely dehydrated, washed twice in 100% butanol (Panreac 14.682.1211) wax embedded (GemCut emerald paraffin, Spiele no. 24364-1), and then sectioned in parallel series (7 μm). All mouse experiments were performed according to the protocols approved by the Universidad Miguel Hernández de Elche OIR Committee (ref. 2014/VSC/PEA/0055).

### *In situ* Hybridization

The technique was realized as previously described (Martinez-Lopez et al., 2015). The RNA probes were obtained from constructions and were kindly provided by Dr. O. Marin (*Robo1* and *Slit2*), Dr. J. Rubenstein (*Dlx2*), and Dr. O. Reiner (*Ntn1*). Each experiment was reproduced five times.

### Immunohistochemistry

The technique was realized as previously described (Martinez-Lopez et al., 2015).

The antibodies used were α-Calbindin (1:100, Swant #CB-48), α-Calretinin (1:200, Swant 7699/4), α-deleted in colon carcinoma (DCC; 1:100, Santa Cruz sc6535), α-ISLET1 (1:100, Hybridoma Bank 39.4D5), and α-NKX2.1 (1:500, Biopat PA 0100). Each experiment was reproduced five times.

### Axonal Tracing

For axonal tracing, embryonic brains or slices were fixed overnight or for 30 min in 4% PFA, respectively. Small DiI crystals (1,1′-dioctadecyl 3,3,3′,3′ tetramethylindocarbocyanine perchlorate; Molecular Probes) or DiA crystals (4-4-dihexadecyl aminostyryl N-methylpyridinium iodide; Molecular Probes) were inserted into the Th of the brains or into the slices previously dissected into 250 μm sections, diffused at 37°C in 4% PFA until the tracers had diffused sufficiently, and then, the brains were cut on a vibratome into 100 μm sections. DAPI (4′, 6-Diamidino-2-Phenylindole, dihydrochloride; Sigma #D9542) was used as a fluorescent nuclear counterstain, diluted in PBS at 0.001%, and incubated 10 min at room temperature. Each experiment was reproduced five times.

### Area Quantification

We have used the measure tool of the Fiji program on a representative section of each experiment. The statistical analysis was realized by an unpaired *t*-test with the GraphPad Prism 8.0.2.

## Results

### Abnormal Thalamocortical Projections in *Gli2* Mutant Mice

**FIGURE 1:**
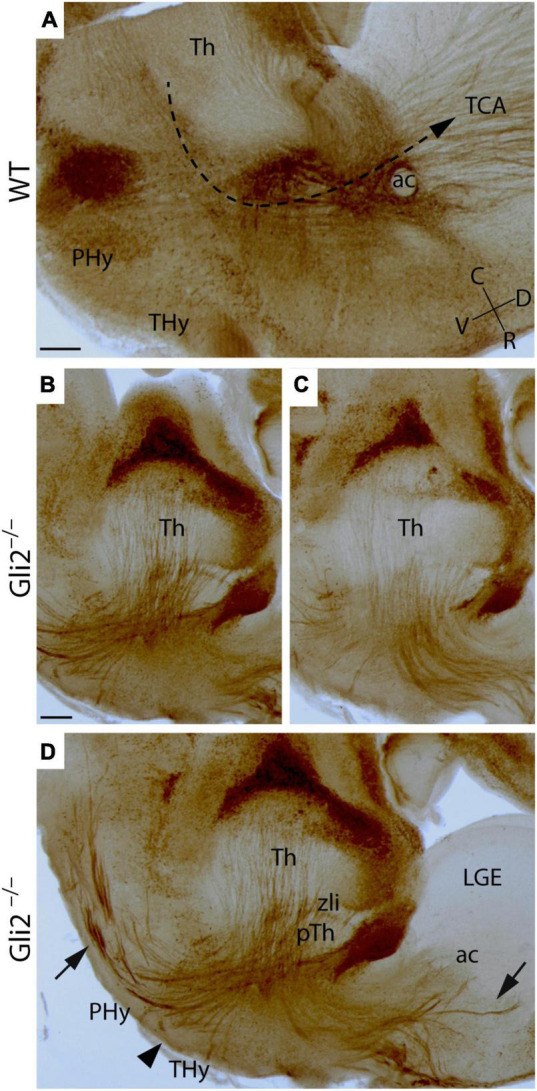
Thalamocortical axonal trajectory in *Gli2* mutant mice embryos. Sagittal sections through wild-type WT **(A)** and *Gli2* mutant **(B–D)** E18.5 embryos with a immunohistochemistry against calretinin. **(D)** Is a merge of images displayed in **(B,C)** to better illustrate the TCAs dispersion. In **(A)**, the dotted arrow indicates the direction of the TCAs trajectory in the WT. The arrows indicate the aberrant trajectory of TCAs to the caudal and rostral region. The arrowhead indicates the axons directed toward the hypothalamus. ac, anterior commissure; C, caudal; D, dorsal; Hy, hypothalamus; LGE, lateral ganglionic eminence; PHy, peduncular hypothalamus; pTh, prethalamus; R, rostral; THy, terminal hypothalamus; TCA, thalamocortical axons; Th, thalamus; V, ventral; zli, zona limitans. Scale bar **(A)**: 300 μm; **(B–D)**: 200 μm.

At E18.5, in the wild type, the TCAs, labeled by the detection of calretinin, are observed to be crossing the diencephalic territory. Once the Phy is reached, they turned dorsally, crossed the alar PHy-telencephalic boundary, and reached the basal ganglia. Finally, they arrived at the Cx, the final destination of the TCAs (Figure 1A). In the mutant embryos, at the same stage, the TCAs displayed a striking abnormal trajectory. Once the axons reached the boundary between the prethalamus (pTh) and the Phy, they appeared strongly defasciculated and were misdirected into aberrant directions (Figures 1B–D). Some TCAs headed toward caudal regions (arrow in Figure 1D), others toward ventral regions of the hypothalamus (Hy) (arrowhead in Figure 1D), and the rest toward rostral regions. Some axons of this last group entered the telencephalic vesicle stopping at the presumptive lateral ganglionic eminence (LGE; arrows in Figure 1D).

**FIGURE 2:**
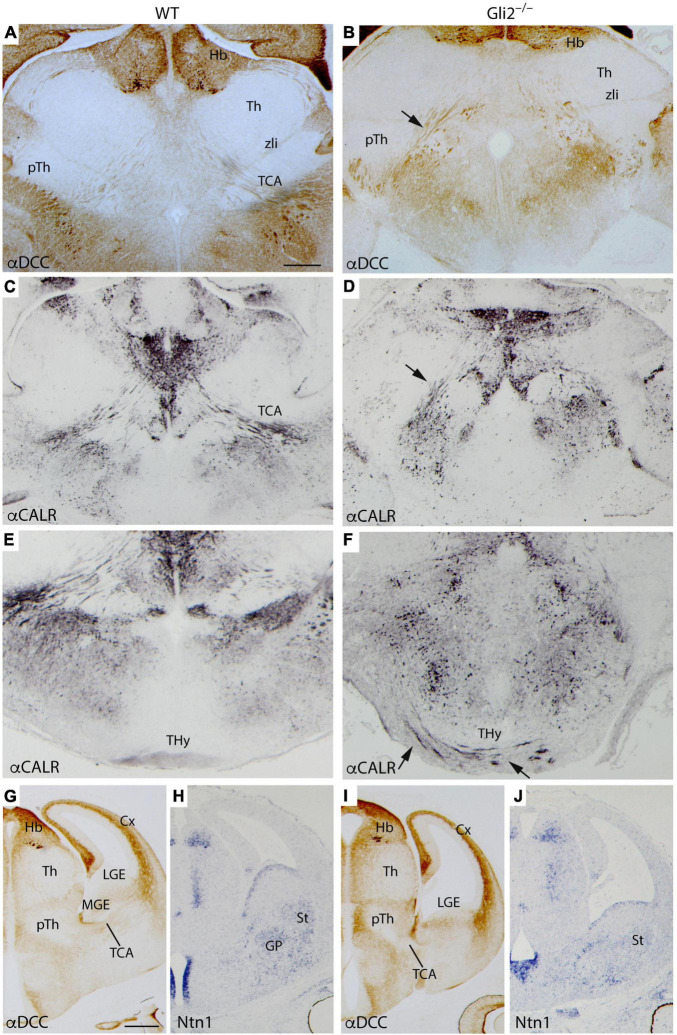
TCAs aberrant trajectory in late- and mid-stage mutant embryos. Selected transversal sections through the forebrain of E18.5 WT **(A,C,E)** and *Gli2* mutant **(B,D,F)** showing immunohistochemistry for α-DCC **(A,B)** revealed with DAB and α-Calr revealed with DAB plus nickel **(C–F)**. The mutant TCAs followed an aberrant trajectory crossing the PHy and the THy [arrow in **(B,D)**] reaching the midline in the basal hypothalamus [arrows in **(F)**]. Coronal sections of E12.5 WT **(G,H)** and *Gli2* mutant **(I,J)** labeled by immunohistochemistry against DCC **(G,I)** and by *in situ* hybridization for *Ntn1* **(H,J)**. Cx, cortex; DCC, Deleted in colon carcinoma; Hb, Habenula; THy, terminal hypothalamus; GP, globus pallidus; LGE, lateral ganglionic eminence; MGE, medial ganglionic eminence; pTh, prethalamus; St, striatum; TCA, thalamocortical axons; Th, thalamus; zli, zona limitans. **(A–F)** Scale bar: 200 μm; **(G–J)** scale bar: 300 μm.

In order to better describe the TCAs anomalous trajectory, we also analyzed mutant and wild-type embryos in a transversal plane of section at the same stage. In the wild type, the DCC (receptor of *Netrin1*) positive axons are detected to be crossing the diencephalic territory through the pTh (Figure 2A). In the mutant brain, the TCAs followed the same route but already displayed some alterations in their distribution (arrow in Figure 2B). In the wild type, once they reached the PHy, following their normal course, they bended dorsally to enter into the telencephalic vesicle (Figure 2C). Meanwhile, in the mutant, the TCAs maintained a straight trajectory toward the hypothalamic territory (arrow in Figure 2D). In the wild type, a section through the basal terminal hypothalamus (THy) confirmed the absence of calretinin positive axons in this territory (Figure 2E). In the mutant, we confirmed the presence of aberrant trajectory and showed its course into the Hy (Figure 2F). The TCAs can be followed into the basal territory of the THy (arrows in Figure 2F). They seemed to reach the ventral midline located in the floor plate. This strong alteration in the TCAs trajectory observed in a late embryonic stage prompted us to analyze mid-stage embryos with an aim to understand the possible causes.

At E14.5 embryos, we labeled transverse sections by immunohistochemistry against DCC and with *Netrin1 in situ* hybridization. *Netrin1-Dcc* signaling mechanism plays an important role in TCAs guidance (Braisted et al., 2000; Powell et al., 2008). The gap of the TCAs was observed already in the corridor of the MGE, crossing the LGE, and reaching the cortex (Figure 2G). The *Ntn1* appeared distributed in the ventricular layer of MGE and LGE and in its mantle layers, globus pallidus in the MGE, and striatum in the LGE (Figure 2H). In the mutant embryos, we found that once the TCAs arrived at the PHy-telencephalic boundary, they were blocked (Figure 2I). The ganglionic eminences also displayed an abnormal morphology. The distribution of *Ntn1* appeared scattered along the mantle layer of the presumptive ganglionic territory (Figure 2J).

**FIGURE 3:**
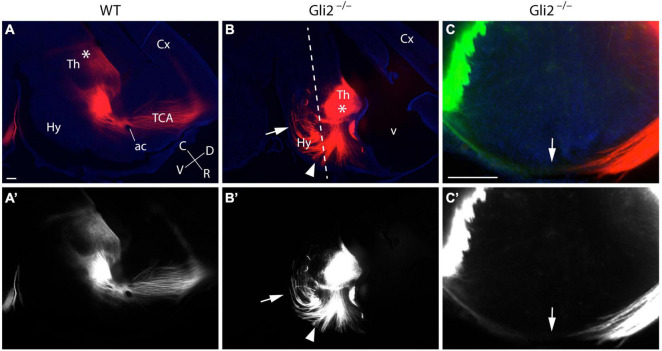
DiI/DiA labeling of thalamocortical axons (TCAs) in Gli2 mutant mice. Selected sagittal vibratome sections (100 μm) through the forebrain of E18.5 embryos WT **(A,A’)** and *Gli2*^–/–^ **(B,B’)** labeled with DiI (red color). Transversal sections **(C,C’)** through the forebrain of E18.5 embryos *Gli2* mutant labeled with DiI (red color) and DiA (green color). DiI implanted in wild-type Th labeled the normal route of the TCAs along the forebrain until they reach the cortex (Cx; **A,A’**). DiI placed in the Th of *Gli2*^–/–^ brains labeled bundles of axons misrouted rostrally, caudally (arrow), and ventrally toward the THy (arrowhead; **B,B’**). Samples sectioned in a transversal plane of section to the hypothalamus [dotted line in **(B)**] to show the final destination of the axons in the Thy, the arrow indicates the floor midline of the Thy **(C,C’)**. Asterisks in **(A,B)** indicate the DiI/A crystal placement sites. The images displayed in **(A–C)** are shown in black and white **(A’–C’)** to facilitate the observation of the labeled axons. ac, anterior commissure; C, caudal; Cx, cortex; D, dorsal; Hy, hypothalamus; R, rostral; TCA, thalamocortical axon; Th, thalamus; V, ventral. Scale bar: 300 μm.

Finally, in order to confirm the thalamic origin of the aberrant axons described in the mutant embryos, we located the crystals of DiI/DiA into the Th of E18.5 *Gli2*^–/–^ mutant and wild-type fixed embryos. In sagittal sections, the DiI crystal located in the wild-type Th allowed us to follow the normal TCAs trajectory. After entering the pTh, they bend dorsally and enter the telencephalic vesicle, where they reach the cortex after they cross the ganglionic eminences (Figures 3A,A’). In the mutant brains, we observed the strong TCAs defasciculation (Figures 3B,B’) and corroborated their aberrant trajectories into caudal and ventral directions (arrow and arrowhead in Figure 3B). The samples sectioned in a transversal plane verified the abnormal TCAs that entered the basal Thy by the application of DiI (red color) and DiA (green color). The left- and right-sided TCAs merged at the THy floor plate (arrow in Figures 3C,C’).

### Morphological Description of Main Forebrain Territories Involved in Thalamocortical Projections in *Gli2* Mutant Mice

We hypothesized that the aberrant trajectory found in the *Gli2*^–/–^ mutant has two possible origins. First, it could be due to an alteration in the specification of the territories crossed by the tract or second, it could be due to an alteration in the signaling mechanisms.

To pursue the identification of the origin of this aberrant trajectory, we analyzed different molecular markers that could allow us to describe the territories related to TCAs guidance.

**FIGURE 4:**
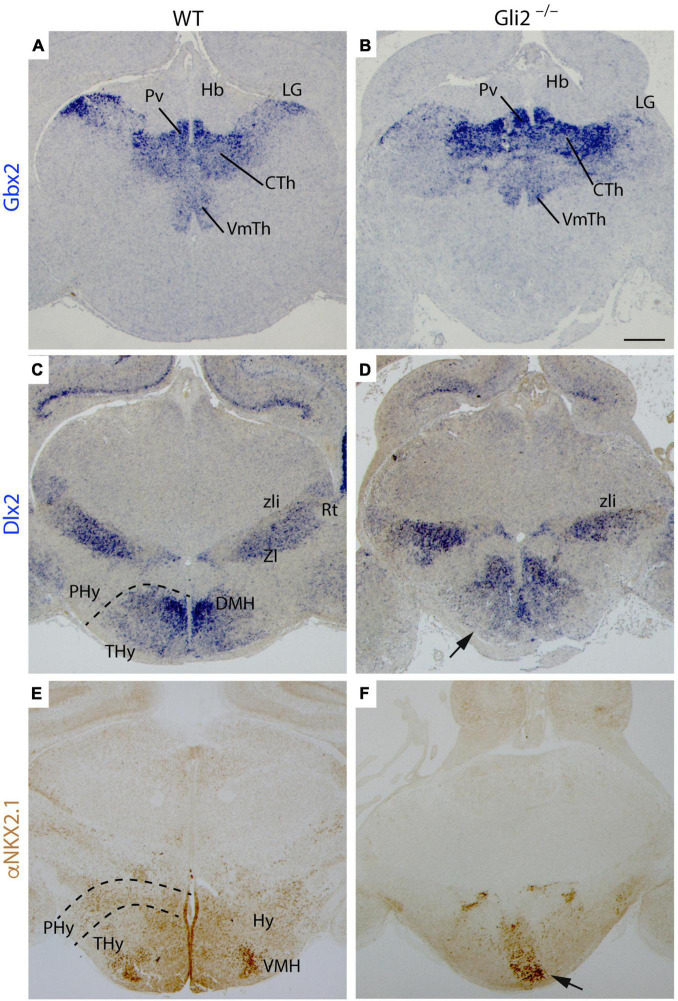
Specification of diencephalon and secondary prosencephalon territories in *Gli2* mutant mice. Frontal sections through wild-type and E18.5 *Gli2*^–/–^ embryos. **(A,B)** Gbx2 *in situ* hybridization. **(C,D)** Dlx2 *in situ* hybridization. **(E,F)** α-NKX2.1 immunohistochemistry in brown. The dotted lines indicate the alar-basal boundary. The Gbx2 expression in the LG, CTH, and PvTh nucleus of *Gli2*^–/–^ thalamus is similar to the one observed in the control **(A,B)**. Dlx2 labels the prethalamic ZI and Rt with a similar pattern than in the control **(C,D)**. Dlx2 is also expressed in DMH in the basal THy of wild type although in *Gli2*^–/–^, this expression was not detected [arrow in **(D)**]. α-NKX2.1 had a scattered distribution in the basal THy, but presented a dense labeling of a VMH subnuclei. This distribution was strongly affected in the mutant [arrow in **(F)**]. CTh, Central thalamus nucleus; DMH, Dorsal medial hypothalamus nucleus; LG, Lateral geniculate nucleus; Hb, Habenula; PHy, peduncular hypothalamus; THy, terminal hypothalamus; Pv, Paraventricular Thalamic nucleus; Rt, reticular nucleus; VMH, Ventromedial hypothalamus nucleus; VmTH, ventromedial thalamic nucleus; ZI, zona incerta; zli, zona limitans. Scale bar: 300 μm.

First of all, the TCAs leave the Th rostrally and cross the pTh in the diencephalon. We studied the expression of *Gbx2*, thalamic-specific homeodomain transcription factor, and *Dlx2*, homeobox gene of the distal-less family expressed in the pTh. In the wild type, we found that *Gbx2* expression was localized in the paraventricular thalamic nucleus, lateral geniculate nucleus, central thalamic nucleus, and ventromedial thalamic nucleus in the Th (Figure 4A). The *Gbx2* expression in *Gli2*^–/–^ Th was slightly abnormal when compared with wild-type embryos (Figure 4B). In the pTh, the *Dlx2* staining was localized in the zona incerta and reticular nucleus in wild-type embryos (Figure 4C). This distribution was similar in the *Gli2*^–/–^ mutants (Figure 4D) with some minor alterations. The areas occupied by the Th and pTh in the mutant did not display a significative difference with the wild type.

Second, the TCAs reached the PHy in the caudal secondary prosencephalon. In this territory, they bend dorsally and reach the telencephalon. Rostrally to the PHy lays the THy, the non-permissive territory for the TCAs. We analyzed *Dlx2*, the homeobox gene of the distal-less family expressed in the basal PHy and THy and NKX2.1 protein distribution (specific marker of both territories). In the wild type, both markers were located in the basal THy. We were able to identify the dorsomedial hypothalamic nucleus and the ventromedial hypothalamic nucleus, being *Dlx2* positive and NKX2.1 positive, respectively (Figures 4C,E). In the *Gli2*^–/–^, the basal THy displayed an aberrant distribution of both markers and we were not able to identify both hypothalamic nuclei (Figures 4D,F). The area occupied by *Dlx2* expression in the mutant was 49% smaller than in the wild type. The comparison of NKX2.1 localization in the mutant displayed a reduction of 66% when compared with the wild type. These differences were statistically significative (*p* < 0.05).

Finally, the TCAs reach the subpallium through the MGE. This is a strongly non-permissive territory for these axons. A group of cells tangentially migrated from the LGE generates a permissive area, known as the corridor that allows the TCAs to cross through this territory (López-Bendito et al., 2006). After their navigation through the LGE, the axons reach their final destination, the cortex.

**FIGURE 5:**
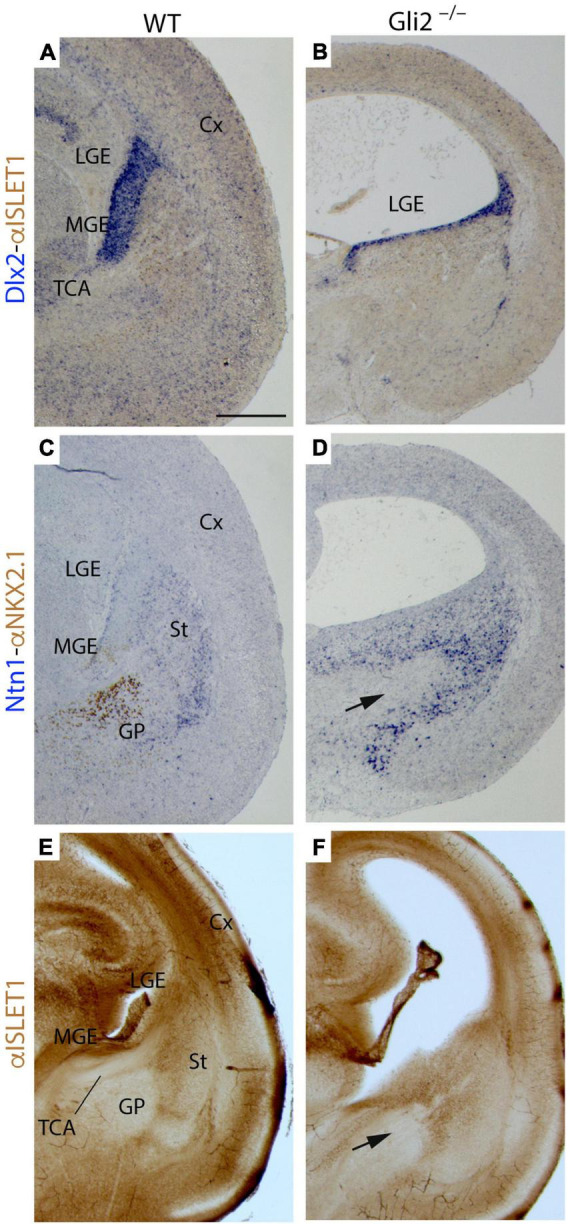
Territories of caudal secondary prosencephalon secondary in *Gli2* mutant mice. Frontal paraffin (7 μm) **(A–D)** and vibratome (100 μm) sections **(E,F)** through wild type and *Gli2*^–^*^/^*^–^ of E18.5 embryos with Dlx2 *in situ* hybridization combined with α-Islet 1 immunoreacted in brown **(A,B)**; Ntn1 *in situ* hybridization combined with α-NKX2.1 immunoreacted in brown **(C,D)**; and α- ISLET1 immunohistochemistry **(E,F)**. In wild type, the ganglionic eminences are differentiated structures (LGE and MGE) but in the mutant, the *Dlx2* expression is reduced and seems to correspond to the LGE **(A,B)**. In the wild type, α-NKX2.1 labels the globus pallidus and ventricular MGE, meanwhile *Netrin1* is expressed in the striatum [LGE mantle layer; **(C)**]. In the mutant, α-NKX2.1 is not detected and there is a wider *Netrin1* positive territory [arrow in **(D)**]. In wild type, α-ISLET1 labels the striatum **(E)** but in *Gli2*^–^*^/^*^–^, these labeling is also expanded [arrow in **(F)**]. Note that the lateral ventricles appeared expanded in the mutant. Cx, Cortex; GE, ganglionic eminences; GP, globus pallidus; LGE, lateral ganglionic eminence; MGE, medial ganglionic eminence; St, striatum; TCA, thalamocortical axon. Scale bar: **(A,C,E)** 200 μm; **(B,D,F)** 300 μm.

With the aim to unveil if these territories are affected in *Gli2* mutant mice, we performed the analysis of *Dlx2* and *Ntn1* mRNA expression combined with the study of NKX2.1 and ISLET1 protein distribution in E18.5 embryos. In the wild type, the *Dlx2* expression domain included the ventricular territory of the MGE and LGE (Figure 5A). In the mutant, the *Dlx2* ventricular expression domain of the presumptive ganglionic eminences was strongly reduced in extension (Figure 5B). In the wild type, the NKX2.1 protein was distributed in the ventricular layer and identified the globus pallidus in the corresponding mantle layer (Figure 5C). In the mutant, we found a complete absence of NKX2.1 protein in the MGE. No positive neurons of the globus pallidus were identified (Figure 5D). The *Ntn1* expression appeared was also affected when compared with the control (Figures 5C,D). In both cases, it labeled the striatal territory in the mantle layer of the LGE. But the distribution in the presumptive mutant ganglionic eminence was clearly affected. A clear gap of *Ntn1* expression was located in this territory, coinciding with the area where the TCAs reach (arrow in Figure 5D). The analysis of ISLET1 protein distribution allowed us to detect the positive cells generated in the LGE that migrated radially to create the striatum. The labeling allowed us to detect the gap generated by the corridor neurons that allowed the TCAs to cross this territory (Figure 5E). In the mutant embryos, the positive cells in the striatum were also detected (Figure 5F). However, the corridor was strongly affected and was much wider than in the wild type (arrow in Figure 5F).

Summarizing, the data mentioned above indicated a strong malformation of the MGE in the mutant embryos. The LGE was also seemed to be affected but its molecular markers were still present. This erroneous specification of the MGE could explain the incapacity of the TCAs to cross this territory and reach the cortex.

### Pathfinding Signaling Alteration in Thalamocortical Projections of the *Gli2* Mutant Mice

Once we analyzed the specification of the different territories involved in the trajectory of the TCAs, we decided to address the second plausible hypothesis for the alterations detected. This is an alteration in the signaling mechanisms needed for the correct wiring of this tract.

**FIGURE 6:**
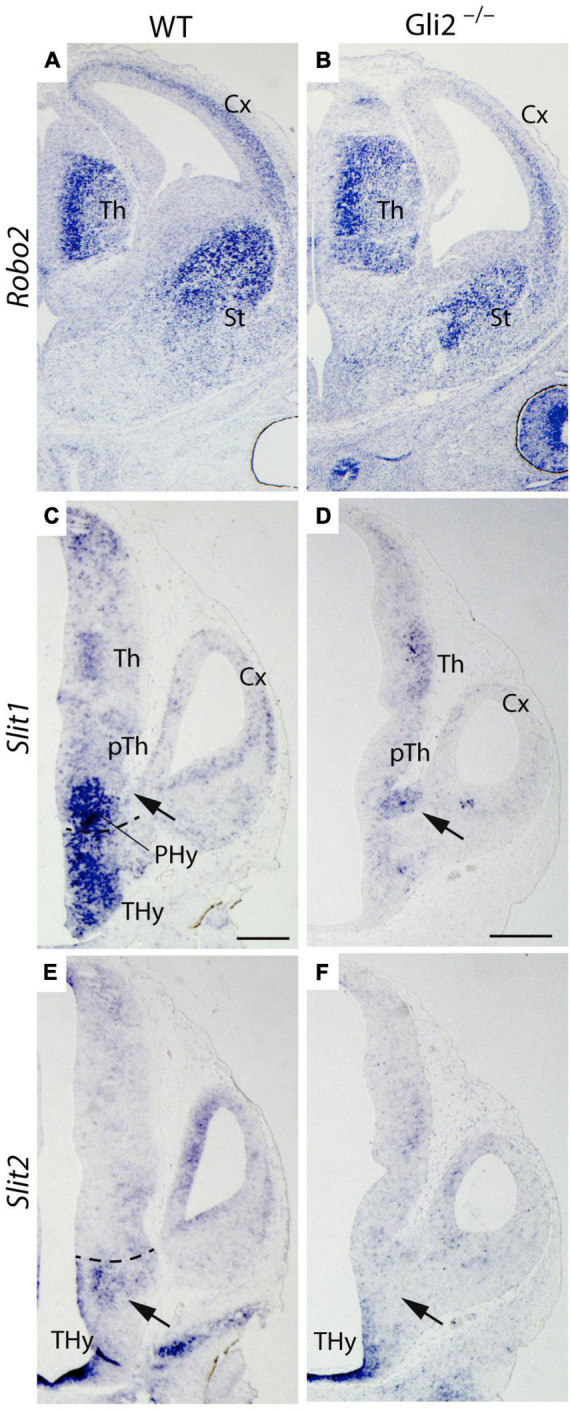
*Robo/Slit* mechanism in TCAs pathfinding in *Gli2* mutant mice. Frontal sections of E14.5 wild-type and *Gli2*^–/–^ brains with Robo2 *in situ* hybridization. Horizontal sections of E12.5 wild-type and *Gli2*^–/–^ embryos with *Slit1* **(C,D)** and *Slit2* **(E,F)** *in situ* hybridization. In **(A,B)**, *Robo2* is detected in the thalamus, striatum, and cortex of wild type and *Gli2*^–/–^. In **(C)**, *Slit1* expression was founded in PHy and THy. A gap of expression is detected in the area where the TCAs cross (arrow). In **(D)**, *Slit1* expression was localized in the zone used by the TCAs to enter the telencephalic vesicle (arrow). In **(E)**, *Slit2* was detected in the midline of the THy and in a domain bordering the limit with the PHy (arrow). In **(F)**, the *Slit2* expression in the mutant appeared only in the THy midline, being absent in the rest of the Thy territory (arrow). The dotted line indicates the limit between Thy and Phy. Cx, Cortex; PHy, peduncular hypothalamus; pTh, prethalamus; St, striatum; Th, thalamus; THy, terminal hypothalamus. Scale bar: 300 μm.

The *Robo-Slits* integrate one of the main chemo-repulsive signal mechanisms involved in axon guidance (Blockus and Chédotal, 2016). It has been directly related to TCAs guidance and their repulsion from the hypothalamic territory (Braisted et al., 1999). *Robo2* receptor expression was detected in the Th of both *Gli2*^–/–^ and wild-type embryos (Figures 6A,B). Therefore, we decided to analyze the expression pattern of *Slit1* and *Slit2*, principal ligands for the *Robo* receptors, in E12.5 *Gli2*^–/–^ and wild-type embryos.

We focus our attention in the PHy, the territory where the axons defasciculate and change their normal direction. The main area of interest is located under the interventricular foramen, the place where the TCAs cross the PHy-telencephalic boundary.

In the wild type, *Slit1* expression was found in a large domain in the PHy and THy. A clear gap of expression was detected in the area where the TCAs cross in order to get into the telencephalon (arrow in Figure 6C). In the mutant, *Slit1* expression was localized in a spot underneath the interventricular foramen (arrow in Figure 6D), a precise location where the TCAs enter the telencephalic vesicle. No expression was detected in any other area of the PHy and THy. Regarding *Slit2* expression, in the wild type, *Slit2* was detected in the THy bordering the PHy (arrow in Figure 6E). In the mutant, it was found in a thin territory at both sides of the THy midline. No expression was detected in other areas of both hypothalamic territories (arrow in Figure 6F). The data obtained allowed us to hypothesize that in the mutant, the expression of *Slit1* underneath the interventricular foramen prevents the TCAs from crossing into the telencephalic vesicle. The lack of *Slit2* expression in the THy allows the TCAs to invade the territory.

## Discussion

Our working hypothesis is based on the role of the *Shh* signaling mechanism through *Gli2* in the pathfinding machinery of the TCAs. The axons are guided by guidance molecules located along specific pathways (Tessier-Lavigne and Goodman, 1996; Dickson, 2002). They travel following a series of distinct steps in which specific guidance cues located at fixed decision points determine their direction. In axon pathfinding, therefore, not only guidance factors are crucial but their precise distribution in time and space constitutes an essential part of the process.

### Aberrant Thalamocortical Axons in *Gli2* Mutant Mice

**FIGURE 7:**
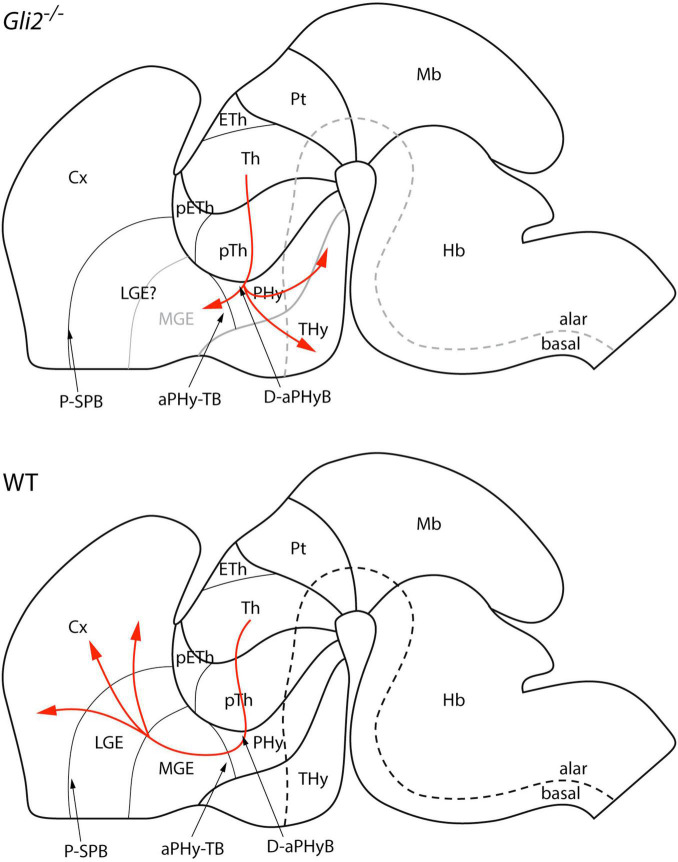
Schematic representation of thalamocortical projections in *Gli2* mutant and wild type in the context of the prosomeric model. aPHy-TB, alar peduncular hypothalamus-telencephalic boundary; Cx, cortex; D-aPHyB, diencephalon alar peduncular hypothalamus boundary; Eth, epithalamus; Hb, hindbrain; LGE, lateral ganglionic eminence; Mb, midbrain; MGE, medial ganglionic eminence; pETh, prethalamic eminence; PHy, peduncular hypothalamus; THy, terminal hypothalamus; P-SPB, pallial-subpallial boundary; Pt, pretectum; pTh, prethalamus; Th, thalamus.

The data obtained pointed out a clear defect in the TCAs trajectory in the loss of the function of *Gli2*. The TCAs are able to leave the Th, cross the pTh, and after entering the Phy, they defasciculate and derail from their normal course. Some axons turn caudalward, others get into the basal Thy, and the rest try to enter unsuccessfully into the telencephalic vesicle. This behavior of the tract implies a complete failure in the signaling mechanisms needed to drive the TCAs into the cortex. The different Th axons are not able to read their way and this lack of information drives them in different directions (almost randomly; Figure 7). Haddad-Tóvolli et al. (2012) described the Th alterations in *Gli2* and *Gli3* loss of function. They briefly described in the *Gli2* mutant some thin and sparse axons in the cortical layers without describing any misrouting of the tract out of the telencephalic vesicle.

We have closely followed the prosomeric model to describe and interpret the pathway of the TCAs in the wild-type and in the *Gli2* mutant. Hence, our interpretation differs slightly from the previous published descriptions (López-Bendito and Molnár, 2003; Garel and López-Bendito, 2014; López-Bendito, 2018; Nakagawa, 2019). First, the trajectory in the diencephalon involves the alar plate of Th and pTh. Second, to enter into the secondary prosencephalon, the TCAs must cross the diencephalic (prethalamic)-secondary prosencephalon (PHy; Figure 7). In this area, the TCAs bend dorsally and get into the telencephalic vesicle (PHy-telencephalic boundary; Figure 7). Finally, the TCAs cross the pallial-subpallial boundary inside the telencephalic vesicle (López-Bendito and Molnár, 2003; Garel and López-Bendito, 2014).

### Specification of the Territories Involved in Thalamocortical Axons Trajectory: Thalamus, Prethalamus, and Secondary Prosencephalon

The specification of two important prosomeres of the diencephalon, Th and pTh, is slightly altered in *Gli2* mutant mice. In fact, the thalamocortical efferents development is a intricated process that depends on the accurate fate specification of thalamic neurons by a cascade of key transcription factor genes (López-Bendito and Molnár, 2003). Furthermore, it is shown that the *Shh* signaling pathway plays a role in the thalamic development (Kiecker and Lumsden, 2004; Vieira et al., 2005; Scholpp and Lumsden, 2010). The GLI transcription factors act downstream of *Shh* and cooperate to integrate *Shh* and other essential morphogenetic signals (Zhou et al., 2004; Kataoka and Shimogori, 2008). While *Gli2* and *Gli3* have transcriptional activator and repressor activities, *Gli1* is mainly a transcriptional activator [reviewed by Ruiz i Altaba et al. (2007), Jetten (2018), and Niewiadomski et al. (2019)].

In the *Gli2 zfd/zfd mutants*, the Th is reduced (mostly in caudal regions) and not completely absent (Haddad-Tóvolli et al., 2012). Apparently, this is due to the *Gli3* activator compensation (*Gli*3A). In the *Gli2* absence, the *Gli3* activate form can be induced by high levels of *Shh* signaling, and in combination with *Gli1A*, it can rescue at least partially the *Gli2* loss of function (Bai et al., 2004). However, *Gli3A* is a weak activator (Dai et al., 1999; Shin et al., 1999; Litingtung and Chiang, 2000; Motoyama et al., 2003; Bai et al., 2004) and therefore, it cannot fully restore the Th proliferation and differentiation.

On the other hand, the pTh is an intermediate target of TCAs (Deng and Elberger, 2003; Molnár et al., 2012). There are evidences that Fgf10 plays an important dose-dependent role as a guidance cue in the pTh to direct the TCAs (Liu et al., 2020). In the *Olig2*-KO mouse or *Rfx3*-KO (ciliopathy) mouse diencephalon, it was described as a prethalamic malformation, followed by disorganized extension of TCAs (Ono et al., 2014; Magnani et al., 2015; Andreu-Cervera et al., 2021). So, the proper formation of the pTh is crucial for the correct extension of TCAs.

The alteration in the specification of the alar diencephalic territories by the loss of function of *Gli*2, therefore in an altered *Shh* signaling cascade, was expected. Being the Th and the pTh alar structures, it is plausible to believe that they would not be strongly affected by the lack of *Shh* signaling from the basal and floor plates. However, these two structures lay at both sides of the zona limitans intrathamica (zli). That is the main secondary organizer of the diencephalon. It secretes SHH as morphogen, so it is reasonable to think that any alteration of this signaling mechanism should strongly affect the patterning of these two structures. The capability of the *Gli* members to compensate each other could explain the weak phenotype of the diencephalon in the loss of function of *Gli2*.

The TCAs leave the pTh to reach the Hy in the caudal secondary prosencephalon. Our results indicate that alar and basal plates of Hy are strongly affected in *Gli2* mutant mice. This altered phenotype sustains the reason for the defasciculation and misdirection of the TCAs as soon as they enter the PHy. One of the possible reasons for this altered course of the TCAs is the incorrect specification of the PHy and THy.

It was demonstrated that the Hy is a repulsive territory for TCAs, avoiding their ventral navigation in their route toward the telencephalon (Braisted et al., 1999). The morphogene Fgf10 has been recently related to this repulsive effect (Liu et al., 2020). Indeed, in *Nkx2.1* mutant, the absence of the basal Hy does not impair thalamocortical projections due to the normal specification of the alar Hy (Marín et al., 2002). In the *Gli2* mutant, the basal Hy is severely altered but not absent, this could explain that in our case, the TCAs are able to find a permissive territory to invade. The alteration of *Gli* genes results in the malformation or absence of the floor plate marked by the *Shh* target gene *Hnf3*β (Matise et al., 1998). Therefore, the *Gli2* absence generates an altered basal plate that probably lacks the correct code of navigating signals.

In fact, mutations in the zebrafish gene *you-too* (*yot*; homologous of mammalian *Gli2*) affected the ventral forebrain patterning (Karlstrom et al., 1999). The alterations described in *yot* mutant embryos unveiled the role of G*li* genes and the Hh signaling pathway in the ventral forebrain cell fate specification. These defects include the alteration of the cellular cues needed to guide the ventral forebrain midline cross by the axons and disrupt optic chiasm formation and optic nerve crossing. The defects described and the *Shh* involvement in human holoprosencephaly (for review, refer Ming and Muenke, 1998) suggest that *Gli2* mutations might be responsible for human diseases such as facial and forebrain midline structures congenital malformations.

At last, some TCAs are able to leave the PHy and enter into the evaginated portion of the telencephalic vesicle. They reach the MGEs, in the presumptive territory of the corridor where they stop their course. We cannot exclude from our experiments that some residual TCAs are able to cross the LGE and finally reach the cortex, agreeing with the conclusions of Haddad-Tóvolli et al. (2012).

The specification of the ganglionic eminences in the *Gli2* mutant is strongly affected, mainly the MGE. This is translated into the incapability of the TCAs to go across this territory. Surprisingly, a medial transformation into LGE with an expansion of the striatum, by the loss of *Nkx2.1* function (Sussel et al., 1999), described the normal generation of the thalamocortical projections (Marín et al., 2002). A reasonable explanation for these two opposite phenotypes is that in the *Nkx2.1* mutant, the LGE is completely unaffected and therefore the needed corridor is correctly formed. In the *Gli2* mutant, we cannot exclude an alteration in the specification of this territory and an aberrant generation of the corridor; in fact, this structure appeared abnormally wide in our mutant. It has been demonstrated that the corridor, migrated cells from the LGE into the MGE, is necessary and sufficient to allow the correct trajectory of the TCAs (Casarosa et al., 1999; López-Bendito et al., 2006).

### Signaling Mechanism Alterations in Thalamocortical Axons Pathfinding in *Gli2* Mutant Mice

The previous studies in *Gli2* mutants have reported errors in longitudinal axonal tract navigation in forebrain, midbrain, and hindbrain (Farmer et al., 2008; Mastick et al., 2010). These included midline crossing, anteroposterior misdirection, and dorsoventral repositioning. Therefore, these diverse phenotypes suggest the interaction of *Gli2* with multiple guidance mechanisms (Farmer et al., 2008; Mastick et al., 2010).

In our mutant, the TCAs follow a normal trajectory until the PHy where they disorganize, misdirect, and grow in all directions. This behavior of the tract indicates an almost complete absence of its signaling sources. This territory of the secondary prosencephalon has severe defaults in its specification and this could therefore indirectly affect its signaling molecules cocktail.

The Th is a heterogeneous group of neuronal populations. Each of them has a unique combination of receptors that will supply them the capability to display different behaviors in front of the same signaling molecules (Leyva-Díaz and López-Bendito, 2013). This fact explains the diversity found in the analysis of our mutants; some TCAs followed a caudal direction, others a ventral direction, and finally some of them were able to get into the MGE.

The *Robo-Slit* repulsive signaling mechanism has been analyzed in the thalamic projections. It plays a pivotal role in the development of these projections, acting at all choice points along its pathway (Braisted et al., 1999; Leyva-Díaz and López-Bendito, 2013; Blockus and Chédotal, 2014). We found alterations in the expression patterns of *Slit1* and *Slit2*, which could explain the TCAs misdirection. A *Slit1* positive patch of expression was detected in the putative area of entrance to the telencephalic vesicle. The TCAs sensible to this molecule were repelled and redirected into caudal and ventral directions. The TCAs non-sensitive to *Slit1* were able to enter into the MGE. The lack of *Slit2* expression in the THy would explain the capability of the TCAs repelled by *Slit1* to invade the Hy. This conclusion is also supported by the analysis of the TCAs trajectory in *Pax6*-KO mice in which their axons are able to reach the hypothalamic territory due to a strong reduction in their levels of *Robo2* in the thalamic neurons (Clegg et al., 2015). A similar result has been recently described by the loss of c-Jun N-terminal kinase (JNK) signaling in the Dlx5/6 territory, the TCAs axons enter in the hypothalamic due to a total failure of the guidance cues and guideposts (Cunningham et al., 2021).

## Conclusion

The alteration of the *Shh* signaling driven by *Gli2* affects the specification of different territories related with the TCAs course. The PHy, Thy, and MGE are strongly affected. The LGE is also altered but in a lower degree, its alteration affects mainly the generation of the corridor. This erroneous specification of the territories produces huge modifications in the pathfinding signaling mechanisms of the TCAs.

## Data Availability Statement

The original contributions presented in the study are included in the article/supplementary material, further inquiries can be directed to the corresponding author.

## Ethics Statement

All mouse experiments were performed according to protocols approved by the Universidad Miguel Hernández de Elche OIR Committee (ref. 2014/VSC/PEA/0055).

## Author Contributions

AC-M, JM-B, and EP conceived and designed the experiments and analyzed the data. AC-M, JM-B, VC, MM, and FA-G performed the experiments. AC-M, JM-B, SM, and EP wrote the article. SM and EP obtained funding. All authors had full access to all the data in the study and took responsibility for the integrity of the data and the accuracy of the data analysis.

## Conflict of Interest

The authors declare that the research was conducted in the absence of any commercial or financial relationships that could be construed as a potential conflict of interest.

## Publisher’s Note

All claims expressed in this article are solely those of the authors and do not necessarily represent those of their affiliated organizations, or those of the publisher, the editors and the reviewers. Any product that may be evaluated in this article, or claim that may be made by its manufacturer, is not guaranteed or endorsed by the publisher.
